# Gene expression profiling of canine osteosarcoma reveals genes associated with short and long survival times

**DOI:** 10.1186/1476-4598-8-72

**Published:** 2009-09-07

**Authors:** Gayathri T Selvarajah, Jolle Kirpensteijn, Monique E van Wolferen, Nagesha AS Rao, Hille Fieten, Jan A Mol

**Affiliations:** 1 Department of Clinical Sciences of Companion Animals, Utrecht University, Yalelaan 108, 3584 CM Utrecht, The Netherlands; 2 Department of Veterinary Clinical Studies, University Putra Malaysia, 43400 UPM Serdang, Malaysia

## Abstract

**Background:**

Gene expression profiling of spontaneous tumors in the dog offers a unique translational opportunity to identify prognostic biomarkers and signaling pathways that are common to both canine and human. Osteosarcoma (OS) accounts for approximately 80% of all malignant bone tumors in the dog. Canine OS are highly comparable with their human counterpart with respect to histology, high metastatic rate and poor long-term survival. This study investigates the prognostic gene profile among thirty-two primary canine OS using canine specific cDNA microarrays representing 20,313 genes to identify genes and cellular signaling pathways associated with survival. This, the first report of its kind in dogs with OS, also demonstrates the advantages of cross-species comparison with human OS.

**Results:**

The 32 tumors were classified into two prognostic groups based on survival time (ST). They were defined as short survivors (dogs with poor prognosis: surviving fewer than 6 months) and long survivors (dogs with better prognosis: surviving 6 months or longer). Fifty-one transcripts were found to be differentially expressed, with common upregulation of these genes in the short survivors. The overexpressed genes in short survivors are associated with possible roles in proliferation, drug resistance or metastasis. Several deregulated pathways identified in the present study, including Wnt signaling, Integrin signaling and Chemokine/cytokine signaling are comparable to the pathway analysis conducted on human OS gene profiles, emphasizing the value of the dog as an excellent model for humans.

**Conclusion:**

A molecular-based method for discrimination of outcome for short and long survivors is useful for future prognostic stratification at initial diagnosis, where genes and pathways associated with cell cycle/proliferation, drug resistance and metastasis could be potential targets for diagnosis and therapy. The similarities between human and canine OS makes the dog a suitable pre-clinical model for future 'novel' therapeutic approaches where the current research has provided new insights on prognostic genes, molecular pathways and mechanisms involved in OS pathogenesis and disease progression.

## Background

Naturally occurring cancer in the dog has been repeatedly emphasized as an excellent model for humans, because similarities in histology, tumor biology, disease progression and response to conventional therapies offer a unique translational opportunity in the broader prospect of cancer research. Since the release of the canine genome in 2005, dog spontaneous tumors have been in the spotlight for 'state-of-the-art' linkage to preclinical human cancer research, where strong similarities in cancer associated gene families were found when comparing the humans to dog [1]. Among the tumors of the dog, osteosarcoma (OS), an aggressive malignant bone tumor that occurs spontaneously, is one of the most outspoken and invaluable cancer for comparative oncology studies [2]. Commonly affected dog breeds include the large-to-giant breeds [3-6]. The median age of dogs affected with OS is around 7 to 10 years, with a subset of tumors arising in younger dogs (18-24 months). The appendicular skeleton is affected in 77% of the dogs, implying an association with rapid early bone growth [3,7] as well as with increased stress on weight bearing areas of the limb. Affected dogs often present with progressive lameness, hard bony swelling or even pathological fracture of the affected bone [8,9]. No strong sex predilection is noted, although males are overrepresented in most studies. Histologically, OS is a heterogeneous tumor that in addition to producing an osteoid matrix, can also present with a fibroblastic and cartilaginous matrix. OS is commonly subdivided into osteoblastic, fibroblastic, telangiectatic, chondroblastic and mixed forms classifications [10].

The prognosis of dogs with OS is unfortunately poor, mainly due to its fast spreading nature; by the time the tumor is found at the primary site, most have already metastasized [11], usually to the lungs, or less frequently in bone and other soft tissues [12]. The goal of therapy is to remove the primary tumor and detectable metastases as well as to initiate multimodal chemotherapy to eradicate micrometastases. The prognosis varies with the type of surgery and chemotherapy [13,14]. The prognosis for dogs without surgery and adjuvant chemotherapy is poor, with a median survival time of 1-3 months. With amputation alone, median survival time can vary from 1 to 6 months [12,15,16]. Some dogs develop metastases within 4 months regardless of the therapy modality, while others survive for longer periods of time [17]. One of the key factors contributing to intensified proliferative activity of the tumor in dogs leading to poor outcome is the deregulation of cellular signals, including growth factors and hormones [18,19]. This poor prognosis feature is comparable to human OS, where there is still ample room for new therapeutics development, primarily to eradicate micrometastases and improve survival.

Recent advances in human cancer management have focused on molecular targeted therapies where high throughput screening technologies have been incorporated to identify novel markers for cancer pathogenesis and specific characterizations of tumors. Over the last decade, gene expression profiling has been able to identify key genes and cellular signaling pathways involved in development and progression of human OS. Microarray technology, a robust method for analyzing global gene expression profiles has been incorporated in those studies. In line with this technology, human OS cell lines have been compared with normal human osteoblasts to provide insights into genes that are involved in OS tumorigenesis [20]. Aside from the conventional comparisons between tumor and normal tissues, other comparisons have been conducted in human OS, including the differential analysis of metastatic tumors compared with less aggressive tumor models [21], gene expression profiling that predicts response to chemotherapy [22] and molecular classification of chemotherapy resistant pediatric OS [23]. The use of these genetic markers for diagnosis and/or prognosis in canine OS is not completely understood and to date no such prognostic global gene profiling have been carried out for the dog.

Thus, for comparative pathobiology and new drug discovery arenas, it would be useful to stratify dogs at diagnosis into 'poor' and 'good' outcome groups based on tumor gene expression profiles. To address this issue, a canine-specific cDNA microarray representing 20,313 genes was used to differentiate the gene expression profile of tumors from dogs that survived less than 6 months from the profiles of those that survived longer. The survival-associated genes and cellular signaling pathways based on global gene expression profiles of thirty-two primary canine OS are identified. Molecular profiling of canine OS with known survival times will help define tumor biology pertaining to prognosis and facilitate future targeted therapies.

## Results

### Retrospective clinical - histological data analysis

**Figure 1:**
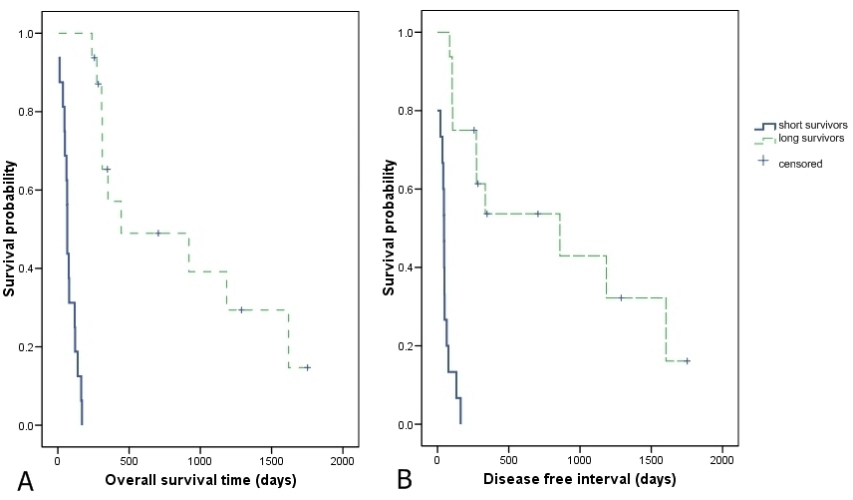
**Kaplan-Meier survival analysis comparing long survivors (LS) and short survivors (SS) of dogs with OS**. (A) Survival time (ST) and (B) disease free interval (DFI) Kaplan-Meier product limit estimate revealed differences in ST with significance of P < 0.0001 (log rank test of 36.58) and DFI with P < 0.0001 (log rank test of 28.35).

The 32 dogs in this study varied in clinicopathological parameters (Table 1). The present experimental design hereby defines dogs with poor prognosis (short survivors, SS) as those surviving less than 6 months and favorable prognosis (long survivors, LS) as those dogs with survival of 6 months or longer. The overall survival times (ST) of these dogs were between 6 and 1752 days with a median value of 204 days. Dogs with poor prognosis (SS) had a ST of 6-169 days (<6 months); while the better prognosis group (LS) had a ST of 239-1752 days (>6 months). Beside the distinction in survival time between these two groups, the SS group also exhibited a significantly shorter disease free interval in a Kaplan-Meier survival analysis (Figure 1). Fisher's exact test revealed no significant differences in discrete data distribution between the 2 groups of survival with respect to sex, neuter status, histological grade and postoperative chemotherapy, the exception being metastatic disease status (P < 0.018). This is because metastatic disease was seen in all dogs in the short survivor's group while 6 dogs from the long survivors' group did not develop metastasis before death (died due to other causes); this was censored for subsequent analyses (Table 2). Tumors were located mostly at appendicular sites, but other locations such as mandible, rib, scapula, metatarsus and extraskeletal were also present. Further potential confounders identified through a univariable Cox regression analysis of the whole population (n = 32) included histological grade, postoperative chemotherapy, alkaline phosphatase (ALP) measurement at diagnosis, sex, age and neuter status (Additional file 1). Alkaline phosphatase (ALP) level and age at presentation were found to have P values of less than 0.15 and were then further subjected to multivariate analysis. Elevation of serum ALP is a known negative prognosticator in dogs with OS and this is apparently also true here, multivariate analysis using the ALP data available for the dogs in this study (n = 23) showed this confounder to be significantly associated with survival (HR = 1.005; CI: 1.000-1.009 with corresponding P value of 0.035).

**Table 1:** Summary of clinical and histology data from 32 dogs used in this study

**SURVIVAL GROUP**	**ARRAY**	**SURVIVAL TIME (days)**	**AGE** **(Year, Month)**	**SEX**	**BREED**	**TUMOR LOCATION**	**HISTOLOGY SUBTYPE**	**HISTOLOGY GRADE**	**POST OPERATIVE CHEMOTHERAPY**
SHORT	1	36	9,2	F	Boxer	Rib	OB/CB/FB	highly malignant	No
SHORT	2	79	8,8	M	Vizsla	distal radius	OB	highly malignant	Yes
SHORT	3	13	2,6	F	Labrador retriever	proximal humerus	OB	highly malignant	No
SHORT	4	169	4,8	M	Great Dane	distal radius	OB/FB	highly malignant	Yes
SHORT	5	121	7,9	M	Rottweiler	proximal humerus	OB/CB/FB	medium malignant	Yes
SHORT	6	66	9,8	M	Dobermann	ulna	OB/TL	medium malignant	No
SHORT	7	119	7,1	F	Rottweiler	distal femur	OB	highly malignant	Yes
SHORT	8	66	11,6	F	Belgian Shepherd (tervuren)	proximal humerus	OB	highly malignant	Yes
SHORT	9	77	7,0	M	Great Dane	distal tibia	OB/FB	highly malignant	Yes
SHORT	10	164	6,7	M	Dobermann	distal tibia	CB	highly malignant	No
SHORT	11	140	7,4	M	Rottweiler	distal radius & ulna	OB/FB	highly malignant	No
SHORT	12	61	5,8	F	Great Dane	distal femur	OB	medium malignant	Yes
SHORT	13	50	11,9	M	Belgian Shepherd (tervuren)	distal tibia & fibula	OB	highly malignant	No
SHORT	14	47	6,8	F	Rhodesian ridgeback	distal femur	OB/FB	highly malignant	Yes
SHORT	15	65	6,2	M	Great Dane	proximal humerus	OB/CB	highly malignant	Yes
SHORT	16	6	6,1	F	Rottweiler	mandible	OB/FB	highly malignant	No
LONG	17	352	9,8	M	Dobermann	proximal tibia	OB/FB	medium malignant	Yes
LONG	18	274	8,8	M	cross	ulna	OB/FB/TL	medium malignant	No
LONG	19	1752	3,8	M	Stabyhoun	extraskeletal	OB/FB	highly malignant	Yes
LONG	20	307	4,3	M	Great Dane	distal radius	CB	medium malignant	Yes
LONG	21	1619	3,7	F	Siberian husky	mandible	OB/TL	highly malignant	No
LONG	22	1185	2,3	F	Flatcoat retriever	rib	OB/TL	highly malignant	No
LONG	23	1289	5,0	F	Rottweiler	distal radius	OB/FB	highly malignant	Yes
LONG	24	257	8,4	F	Bouvier	distal radius	OB/FB	highly malignant	Yes
LONG	25	445	10,4	M	Scottish collie	distal radius	OB	highly malignant	Yes
LONG	26	284	7,0	F	cross	distal radius	OB/CB	highly malignant	Yes
LONG	27	239	8,4	M	Rottweiler	scapula	OB/FB	highly malignant	Yes
LONG	28	705	8,3	F	cross	metatarsus	FB	low malignant	Yes
LONG	29	348	9,0	F	Mastiff	distal radius	OB	highly malignant	Yes
LONG	30	312	5,8	M	Mastiff	scapula	OB/FB	highly malignant	Yes
LONG	31	920	7,3	M	Belgian Shepherd (malinois)	mandible	OB/FB/CB	highly malignant	No
LONG	32	312	10,2	M	Rottweiler	distal femur	OB/CB	highly malignant	Yes

*Abbreviations*: M, male; F, female; OB, osteoblastic; CB, chondroblastic; FB, fibroblastic; TL, telangiectic; highly malignant, Grade 3; medium malignant, Grade 2; low malignant, Grade 1.

**Table 2:** Distribution of variables between the SS and LS groups by Fisher's exact test

**Parameter**	**Short survivors**	**Long survivors**	**(Fisher's exact test)** **P value**
		
	**n**	**n**	
**Gender**			> 0.999
Male	9	9	
Female	7	7	
**Neuter status**			> 0.999
Neutered	5	6	
Intact	11	10	
**Metastasis present at time of death**			0.018**
Yes	16	10	
No	0	6	
**Postoperative chemotherapy**			0.458
No	7	4	
Yes	9	12	
**Histological grade**			> 0.999
Low and medium	3	4	
High	13	12	

Analysis on discrete variables comparing the short and long survivors revealed no significant differences in the distribution of variables assessed except for metastatic disease status. **Significance defined as P < 0.05.

**Figure 2:**
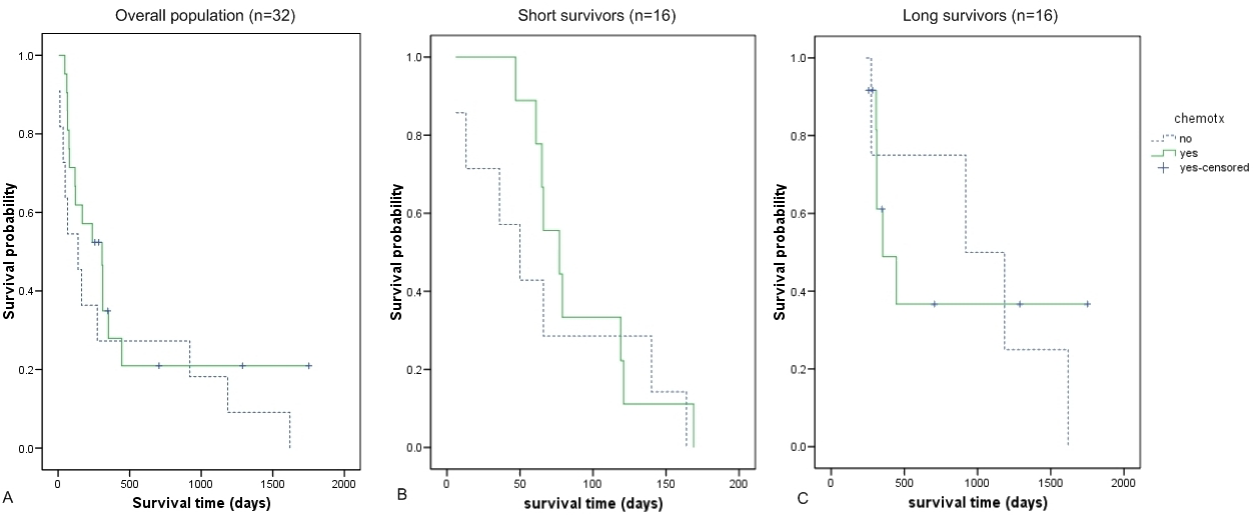
**Kaplan-Meier survival curves of dogs treated with and without postoperative chemotherapy**. (A) Overall population (n = 32 dogs); (B) Among short survivors group (n = 16 dogs) and (C) long survivors (n = 16 dogs). Chemotherapy did not significantly prolong life in the overall population or the subpopulations.

Although the small population size in the present study is unlikely to show significant differences, it is important to identify any possible confounding factors that may influence survival/prognosis and should be considered when interpreting the expression data at biological levels. In both survival groups, dogs were subjected to a variety of single agent therapies, including lobaplatin, doxorubicin or carboplatin; or a combination of carboplatin and doxorubicin. Some were treated with postoperative chemotherapy and some not, and Kaplan-Meier analysis showed that chemotherapy did not significantly prolong survival in the overall population (log rank score 1.353, P value 0.245), neither among short survivors (log rank score 0.460, P value of 0.498) nor among long survivors (log rank score 0.033, P value 0.857) separately (Figure 2). Although these survival curves only represent bivariable analyses (treated vs. not treated) that do not include all confounding variables, a separate univariable and subsequent multivariable statistical model with inclusion of variables such as histological grade, alkaline phosphatase, neuter status, gender and age upon chemotherapy stratification revealed that none of the variables appeared to have influenced survival significantly (Additional file 2). An additional important insight from the long survival group is that all 4 dogs that were censored from the study (death due to other causes and no apparent metastatic disease until death) received postoperative chemotherapy, which suggests possibilities that these dogs had responded reasonably well to the chemotherapeutic regime. Finally, since both survival groups consist of comparable heterogeneous populations of dogs and poor survivors tend to have higher ALP measurements, the arbitrary but defendable approach to a binary distinction of 'good' and 'poor' outcome based on 6 month survival time is supported for further gene expression profiling.

### Microarray data analysis

**Figure 3:**
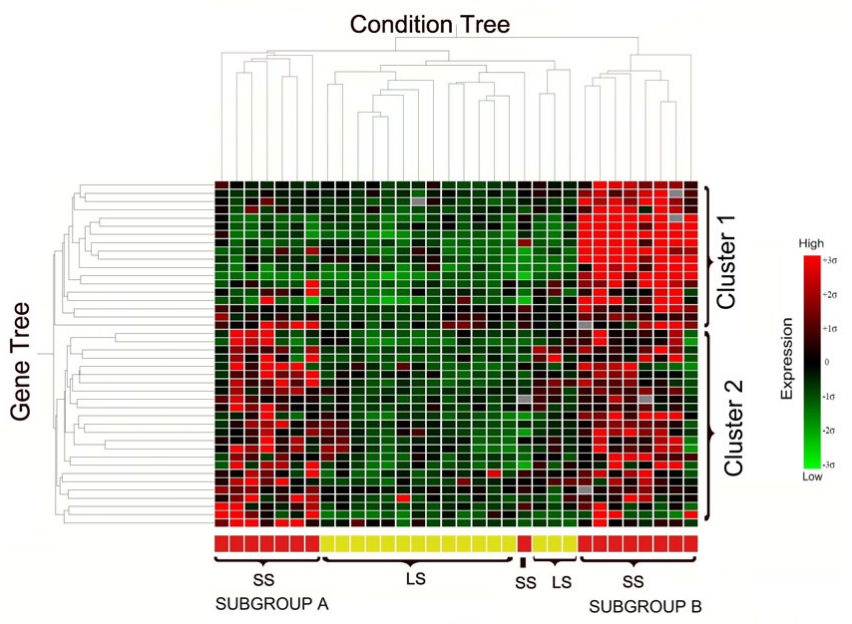
**Dendrogram and heat map generated by hierarchical clustering of differentially expressed genes between SS and LS**. Differentially expressed genes were selected at false discovery rate of 10% between short survivors (SS) and long survivors (LS) of canine OS. Genes found upregulated are shown in red and downregulated genes are represented in green.

**Figure 4:**
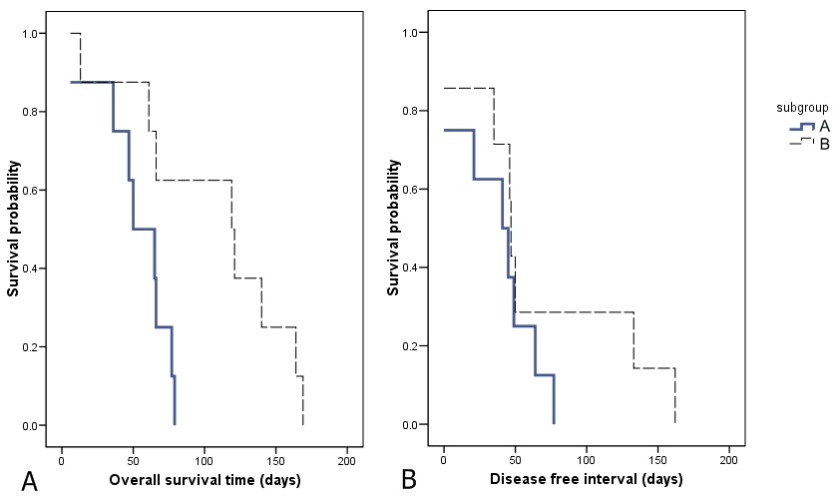
**Kaplan-Meier curves of overall survival and disease free interval comparing subgroup A with subgroup B**. All dogs that lived less than 6 months are characterized as poor survivors but among these dogs there were differences in gene expression which divides them into 2 different subgroups where they were subsequently found to significantly differ in survival time and not disease free interval.

Comparison of short and long survivors using two-class unpaired *Significance Analysis of Microarrays *(SAM) revealed fifty-one genes that were differentially regulated at a false discovery rate (FDR) of 10%. All these genes were found to be upregulated in the SS group where 37 of them were upregulated with a fold change of more than 1.4 (Table 3). Of the 37 genes, 8 were not functionally annotated for the dog and 5 others were unknown Expressed Sequence Tags (ESTs). To further visualize the gene expression data, hierarchical clustering was performed on all 32 arrays based on the differentially expressed genes (Figure 3). A gene tree dendrogram revealed two distinct clusters, where Cluster 2 appears to distinguish long from short survivors or, in other words, this signature defines prognosis based on the 6 month survival binary outcome. Within Cluster 1, particular attention should also be given to a further separation into 2 different subgroups (A and B). Although Fisher's exact test revealed no significant differences among the variables assessed with respect to histological grade, sex, neuter status and postoperative chemotherapy (Additional file 3), this could primarily be due to the low number of subjects within each subgroup (n = 8) which most likely will not allow statistical significance. We did find a significant difference in terms of overall survival time between the two subgroups, with subgroup A: having a shorter survival time than subgroup B. The log rank score was 5.82, with a corresponding P value of 0.0158 (Figure 4).

**Table 3:** Thirty-seven differentially expressed genes between (SS) and (LS) of canine OS based on SAM analysis

**Gene Clone ID**	**Q-value (%)**	**Fold change**	**Gene description**	**Gene symbol**
DG2-21g13	0.00	4.1	WD repeat and SOCS box containing protein 2	WSB2
DG2-23c15	0.00	3.3	cofilin 2	CFL2
DG2-72g4	0.00	2.7	ankyrin repeat domain protein 17 isoform a	ANKRD17
DG42-128j23	0.00	2.1	paraoxonase 1	PON1
DG2-112n11	0.00	2.1	Kinesin heavy chain (Ubiquitous kinesin heavy chain)	UKHC
DG32-161c11	0.00	2.0	WNK lysine deficient protein kinase 1	WNK1
DG2-63l7	0.00	1.7	nuclear receptor co-repressor 1	NCOR1
DG32-237k11	0.00	1.6	Ribosomal L1 domain containing protein 1(PBK1)	RSL1D1
DG11-239n21	0.00	1.6	cell-cycle and apoptosis regulatory protein 1	CCAR1
DG2-28n13	0.00	1.5	28S ribosomal protein S31, mitochondrial precursor	MRPS31
DG2-59p21	0.00	1.4	#NA	
DG2-90b10	4.61	2.2	Heat shock protein HSP 90-alpha	HSP90
DG2-123a3	4.61	2.0	Microsomal glutathione S-transferase 1	MGST1
DG2-86b3	4.61	1.6	Canis familiaris similar to T06D8.1a	
DG8-102i3	4.61	1.5	#NA	
DG2-100e22	4.61	1.5	COMM domain containing protein 8	COMMD8
DG2-106f2	4.61	1.4	Translocation protein SEC63 homolog	SEC63
DG2-130m14	4.61	1.4	Canis familiaris similar to CG1218-PA	
DG2-90c16	6.15	2.6	#NA	
DG43-1a15	6.15	2.4	#NA	
DG14-71c7	6.15	1.8	serine/arginine repetitive matrix 1	SRRM1
DG2-72p3	6.15	1.6	Vacuolar ATP synthase subunit C	V-ATPase C
DG32-216j13	6.15	1.6	Flavin reductase (NADPH-dependent diaphorase) (FLR)	BVRB
DG2-94j4	6.15	1.5	Canis lupus familiaris high-mobility group box 1	HMGB1
DG2-42j4	6.15	1.4	FRA10AC1 protein isoform FRA10AC1-1)	
DG42-89n19	6.15	1.4	#NA	
DG2-19i16	9.70	7.2	#NA	
DG2-18m22	9.70	4.6	plasma glutamate carboxypeptidase	PGCP
DG9-134g22	9.70	1.9	ankyrin repeat domain 11	ANKRD11
DG2-123m9	9.70	1.9	CG12795-PA	
DG14-14i19	9.70	1.7	Stress-70 protein, mitochondrial precursor (HSAP9) (Mortalin)	MOT
DG9-212f9	9.70	1.5	CG12795-PA	
DG2-60f11	9.70	1.5	#NA	
DG2-25k5	9.70	1.4	SMC6 protein	SMC6
DG14-86l17	9.70	1.4	#NA	
DG2-24i24	9.70	1.4	60 kDa heat shock protein, mitochondrial precursor	HSP60
DG11-243e16	9.70	1.4	splicing factor, arginine/serine-rich 2, interacting protein	SFRS2IP

All these genes were upregulated in short survivors. Genes were selected at a FDR <10% and of fold change of ≥1.4. (*#NA: non-annotated for *Canis familiaris*)

### Quantitative real-time PCR analysis

To verify the microarray analysis, QPCR was performed on four candidate genes and two reference genes, HPRT and RPS19 that were used to normalize the expression data. The overall expression patterns of the candidate genes were comparable to the microarray analysis. Quantitative real-time PCR revealed common overexpression of ANKRD17, MGST1, MRPS31 and NCOR1 in short survivors compared to long survivors by 1.35, 1.46, 1.53 and 1.1 fold respectively.

### Molecular functions and biological process analyses

Various molecular functions of the genes differentially expressed by short and long survivors of canine OS were defined into categories according to the Gene Ontology database, including: DNA repair and integrity, cell cycle/proliferation, stress response, apoptosis regulation, protein modification and metabolism, and mRNA transcription regulation (Additional file 4). Several of these genes have been implicated in tumorigenesis and cancer biology for both humans and dogs. HSP70/MOT, HSP60 and NCOR1 have been implicated in human OS, while HMGB1, HSP90 and MGST1 have been associated with both human and canine OS. Generally, the overexpressed genes were linked to 3 main biological processes known in advanced cancer: mainly cell cycle and proliferation, followed by drug resistance and/or metastasis (Table 4).

**Table 4:** Gene transcripts overexpressed in short survivors of canine OS found associated with 3 main biological processes

**Gene ID**	**Fold change**	**Gene description**	**Gene symbol**
**Metastasis-associated**			
DG2-23c15	3.3	cofilin 2	CFL2
DG14-14i19	1.7	Stress-70 protein, mitochondrial precursor (HSAP9) (GRP 75) (Mortalin)	MOT
DG2-72p3	1.6	Vacuolar ATP synthase subunit C	V-ATPase C
			
**Drug resistance**			
DG2-112n11	2.1	Kinesin heavy chain (Ubiquitous kinesin heavy chain)	UKHC
DG2-123a3	2.0	Microsomal glutathione S-transferase 1	MGST1
DG2-72p3	1.6	Vacuolar ATP synthase subunit C	V-ATPase C
DG2-94j4	1.5	high-mobility group box 1	HMGB1
			
**Cell cycle/proliferation**			
DG2-23c15	3.3	cofilin 2	CFL2
DG32-161c11	2.0	WNK lysine deficient protein kinase 1	WNK1
DG14-71c7	1.8	serine/arginine repetitive matrix 1	SRRM1
DG14-14i19	1.7	Stress-70 protein, mitochondrial precursor (HSAP9) (GRP 75) (Mortalin)	MOT
DG32-237k11	1.6	Ribosomal L1 domain containing protein 1(PBK1 protein)	RSL1D1
DG11-239n21	1.6	cell-cycle and apoptosis regulatory protein 1	CCAR1
DG2-106f2	1.4	Translocation protein SEC63 homolog	SEC63
DG2-25k5	1.4	SMC6 protein	SMC6

The 3 processes commonly confer for the aggressive phenotype of tumor in general which include increased drug resistance, high proliferation and differentiation and high metastasis.

### Pathway analyses

**Figure 5:**
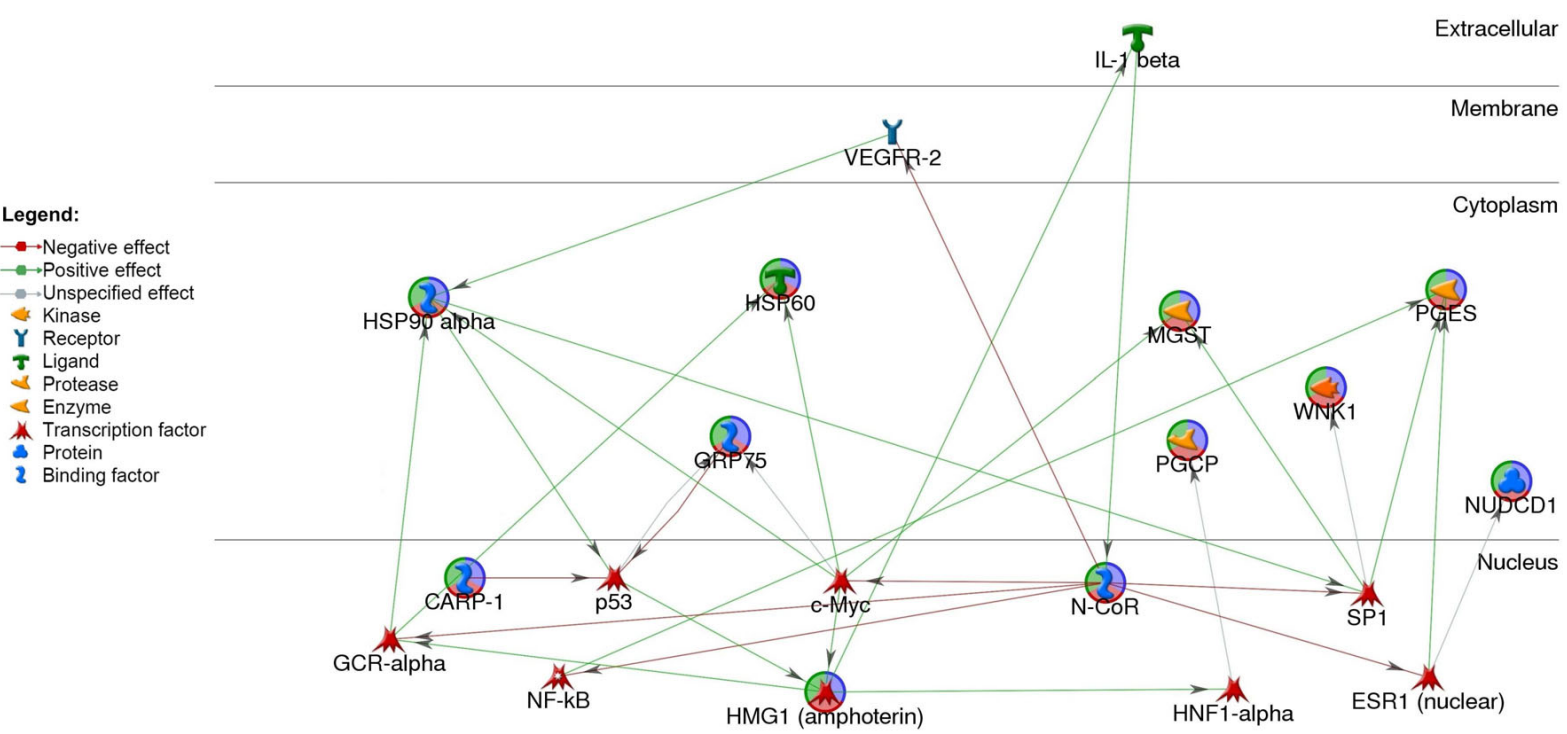
**Gene regulatory network generated through MetaCore's 'shortest path analysis' algorithm**.

The number of annotated genes in the present study is too low to form any relevant protein networks. Therefore, we used the 'Shortest path analyses' by MetaCore, which allows for one intermediate gene to be linked to the differential genes in order to form a more informative gene-protein network. These intermediate genes may be a transcription factor, receptor or ligand. Transcripts with limited information available on network or associated with a pathway were excluded, leaving a final gene regulatory network generated based on 11 genes that were overexpressed in short survivors of canine OS with the addition of seven transcription factors, one receptor and a ligand (Figure 5). Transcription factors p53, c-myc, SP1 and NF-kB seemed to play roles as 'central hubs' connecting these transcripts. All three heat shock proteins (HSP90-alpha, HSP60 and MOT/HSAP9/GRP75) appeared to be linked to a common transcription factor c-myc.

Because the interaction among genes is definitely more complex, we performed a PANTHER^® ^pathway analysis in which functional and pathway data available on human transcripts were used as reference to analyze the transcripts from the present study in the dog. PANTHER^® ^analysis on the 51 differentially expressed genes by SAM yields too few genes associated with a cellular signaling pathway, undermining the power of pathway analysis. This is mainly because a majority of genes are not associated with a signaling pathway, but also because the dog lacks functional annotation at present. Therefore, we excluded the FDR correction for multiple testing and selected a longer list of genes with a threshold of P < 0.05 from EDGE (a microarray analyzing software). After removal of non-annotated genes and transcript replicates, there were 533 genes mapped on PANTHER^® ^which permitted further analysis. Identified pathways included the top four commonly associated with cancer and OS pathogenesis: Wnt signaling, inflammation mediated by chemokine and cytokine signaling, integrin signaling, and ubiquitin proteasome (Table 5). Our pathway list was then compared with three microarray datasets of human osteosarcoma [24,25]. Two gene lists were obtained from Srivastava A., *et al*., 2006: one of genes overexpressed in human osteogenic sarcomas in comparison to normal bones, the other a partial list of genes upregulated in a highly metastatic human OS cell line, and a gene list from Rajkumar T., *et al*., 2008 identifying genes that were differentially expressed in a doxorubicin drug resistant human OS cell line. These gene lists, obtained either from the authors themselves or via the supplementary tables provided in their articles, were subsequently subjected to PANTHER^® ^pathway analysis as similarly conducted in the present study to define pathways pertaining to drug resistance/metastasis/tumorigenesis of OS. Comparison of the top 20 pathway hits from these 3 studies in human OS (Table 5) revealed seven pathways overlapping with the present study on canine OS, including Wnt, chemokine/cytokine, Alzheimer disease-presenilin pathway, fibroblast growth factor (FGF), platelet derived growth factor (PDGF), apoptosis and interleukin signaling pathways.

**Table 5:** Deregulated pathways in canine OS pertaining to aggressive phenotype

**Reference**	**Present study on canine osa**	**Srivastava *et al.*, 2006**	**Rajkumar, T. *et al*., 2008**	**Srivastava *et al.*, 2006**
**Study approach**	**poor survival associated profile**	**high metastatic profile**	**drug resistant profile**	**osteogenic tumor profile**
**Samples used**	**32 primary tumors**	**human osa cell lines**	**cell line resistant to doxorubicin**	**10 tumors and 8 normal bones**
Total differential expressed genes	1426	1999	485	194
Total Mapped IDs on PANTHER^®^	533	1185	295	159

Huntington disease	12	▲	▲	
**Wnt signaling pathway**	**10**	▲	▲	▲
**Chemokine/cytokine signaling pathway**	**8**	▲	▲	▲
Integrin signalling pathway	7	▲		▲
Parkinson disease	6		▲	▲
Ubiquitin proteasome pathway	6		▲	
**Alzheimer disease-presenilin pathway**	**5**	▲	▲	▲
Cadherin signaling pathway	5		▲	
Endothelin signaling pathway	5			
Glycolysis	5			
Heterotrimeric G-protein signaling pathway	4	▲		
Angiogenesis	3			▲
Cytoskeletal regulation by Rho GTPase	3	▲		
EGF receptor signaling pathway	3	▲		
**FGF signaling pathway**	**3**	▲	▲	▲
p53 pathway	3		▲	
**PDGF signaling pathway**	**3**	▲	▲	▲
T cell activation	3	▲	▲	
TGF-beta signaling pathway	3	▲		▲
Androgen/estrogene/progesterone biosynthesis	2			
**Apoptosis signaling pathway**	**2**	▲	▲	▲
B cell activation	2			
Hedgehog signaling pathway	2			
Hypoxia response via HIF activation	2			
Interferon-gamma signaling pathway	2			
**Interleukin signaling pathway**	**2**	▲	▲	▲
Oxidative stress response	2			
p53 pathway feedback loops 2	2		▲	
PI3 kinase pathway	2	▲	▲	
Ras Pathway	2	▲		▲

Comparison of the top 20 pathways from 3 published human OS studies revealed 7 pathways (in bold letters) that are commonly deregulated in both human and canine OS. (▲: Common pathway hits found across different gene sets published for human OS to the present study in canine)

## Discussion

### Canine OS gene expression profiling as a model for human OS

Osteosarcoma (OS) is a devastating disease in both human and dogs. The clinical presentation, characteristics and disease progression is similar in people and dogs except for the age of clinical onset, where 75% of human cases affect young adolescents while onset is predominantly observed in middle-aged to older dogs. For dogs, the decision to initiate treatment is often made by the owners. The standard therapy for canine OS primarily involves amputation or limb sparing surgery, followed by adjuvant chemotherapy. Some dogs tend to develop metastases within 4 months regardless of the therapy modality, while others survive for longer periods of time [17]. Similarly, despite the advance standard of care for children with OS, only 60% reach a 5-year disease free interval and 20% will not survive beyond 5 years [26].

The similarities seen in OS disease progression and survival rates in humans and dogs provides a reasonable justification to compare gene expression profiles based on clinical outcomes once these are adjusted for comparative lifetimes. The similarities in the genetic expression and biological behavior of canine and human OS also makes the dog a suitable model to study this disease [27,28]. Gene expression profiling of spontaneous tumors in the dog offers a unique translational opportunity to identify prognostic biomarkers and signaling pathways common to both species. This is further supported by a report on the strong similarity in the gene expression profile found in both canine and human pediatric OS, suggesting that specific genes and pathways are commonly involved in these two species (Paoloni, M. *et al*., 2005, personal communication).

Further, various clinical and pathological methods are being used as prognostic indicators for canine OS, but to our knowledge, this is the first research conducted using canine specific cDNA microarray to analyze gene expression profiles associated with survival in a panel of thirty-two primary canine OS. The ability to identify gene markers of tumor aggressiveness at the time of primary tumor removal would provide prognostic values for a tailored therapy which can be translated to human medicine.

### Clinicopathological relevance for OS prognostic gene expression studies in dogs

**Figure 6:**
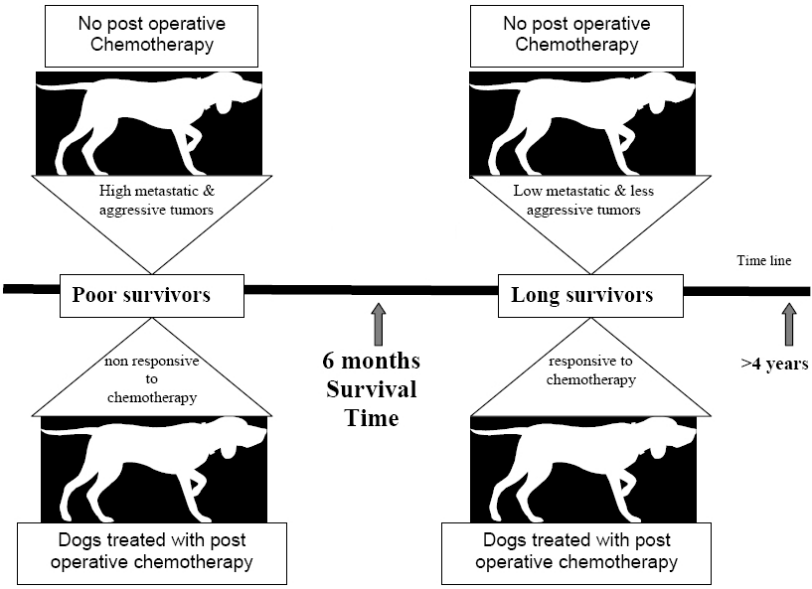
**Classification of dogs: poor survivors (SS) and long survivors (LS)**.

Gene expression profiling on the basis of segregating and then identifying poor and favorable outcome markers is not novel, but this is the first study conducted in dogs with OS. Selection of appropriate tumor candidates based on clinicopathological relevance for gene profiling is crucial for the overall experimental design. Although advanced histological grade and postoperative chemotherapy are known confounders to influence survival and prognosis in dogs with OS [29], these factors lack to significantly influence survival in the present study population, mainly due to the small population size. Elevation of serum alkaline phosphatase is a known negative prognosticator in dogs with OS, which is true for the present study population where poor survivors had significantly higher alkaline phosphatase levels than long survivors. Some dogs, despite receiving postoperative chemotherapy, did not live beyond 6 months and can be characterized as 'poor responders' to chemotherapy. On the other hand, the majority of dogs that received chemotherapy within the long survivors was censored which suggests that either these tumors responded to the chemotherapy or the primary tumor did not harbor the aggressive phenotype. These data further justify distinguishing between good and poor outcome: dogs can be assessed at the gene-biological level of primary tumors regardless of post-operative chemotherapy. In addition, the current gene profiling was carried out on tumors prior to chemotherapy, where 'clues' at gene expression levels will help to determine which animals will probably live longer regardless of either the type of regime or decision to include postoperative chemotherapy (Figure 6). To further improve the efficacy of therapy, it is necessary to identify dogs with OS with an increased risk of treatment failure, as well as to identify those that might not need aggressive chemotherapeutic protocols.

To address issues concerning applicability for tailored therapies, microarray technologies have identified new molecular subclassifications in various human tumors, but to date there are no such reports for prognostic signatures in human and canine OS. The current research thus provides new insights into possible prognostic molecular subclassifications in canine OS. Hierarchical clustering of the differentially expressed genes revealed that long survivors have a similar gene expression profile, which is distinctly different from that of short survivors: these genes appear to be consistently downregulated among the long survivors. In contrast, clustering the genes based on expression was found to separate the short survivors into 2 subgroups. Cluster 1 genes were found to be overexpressed in subgroup B in comparison to the rest of the tumors, making this subgroup unique among the 32 tumors. Intriguingly, subgroup A appeared to have a significantly decreased survival compared to subgroup B where it shares common downregulation of genes with cluster 1. The mechanism behind this evident contradiction can only be speculated. Possibly, upregulation of both cluster 1 and cluster 2 genes provides a somewhat protective mechanism that improves survival as compared to the combination seen for subgroup A. On the other hand, the data can also be interpreted such that Cluster 1 genes are upregulated in 25% of canine OS, where its overexpression is related to poor prognosis. Cluster 2 genes appear to be true prognostic gene signature, defining the gene expression that segregates short (<6 months) from long survivors. The importance of these findings is they provide insights into possible existence of molecular subclassifications among short survivors that may be regulated by different signaling pathways whereby, due to the complex interaction among genes, these subgroups may respond differently to certain targeted therapies. These interpretations must be addressed cautiously given the small number of samples and differentially expressed genes selected for the generation of the present dendrogram. As has been noted previously, "a replicable classification is not necessarily a useful one; but a useful one that characterizes some aspect of the population must be replicable" [30]. Therefore, insights into molecular sub-classifications among the canine OS from the present study require further validation on a larger and independent set of tumors.

### Differentially expressed genes between short and long survivors: similarities with human OS

For a more biologically meaningful approach to data analysis, we looked at the level of common biological processes that differentiates short from long survivors. Genes highly expressed among short survivors of canine OS are likely to contribute to the aggressive nature of the disease in terms of increased cell cycle and proliferation, drug resistance and metastasis-associated properties. Among the transcripts that were differentially expressed between the two survival groups that have been implicated in human OS were HSP70/MOT, HSP60 and NCOR1, while HMGB1, HSP90 and MGST1 have been reported for both human and canine OS. Following discussion will focus on the relevance of these genes to human and canine OS pathogenesis and disease progression.

The main goal for discovering new therapeutic interventions for OS is to inhibit metastasis and eradicate micrometastases. The difference in growth patterns of metastases may be detected by differences in gene expression of primary tumors at the point of amputation. Due to their gene expression differences, some dogs develop metastasis rapidly and others have delayed metastatic disease, which contributes significantly to their survival. In the overall clinical data comparison in this study, there were long survivors who did not develop metastases at all. We did not exclude these tumors from the analysis because they appear to be true survivors, where there was no metastatic disease detected after primary tumor removal. Among the differentially expressed genes between short and long survivors, CFL2, MOT and V-ATPase C have been associated with invasion and metastasis in various human tumors [31-33]. The metastatic potential of the stated genes in OS has not been reported previously; therefore additional research into their exact functions in OS metastasis is required.

Over the past decade, besides metastasis, there has been growing interest in the development of multidrug resistance in OS, where various genes have been reported to contribute to the drug resistance phenotype [24,34,35]. The two drug resistance related genes overexpressed in poor survivors found in the present research in canine OS include HMGB1 and MGST1. HMGB1 expression levels have been previously investigated in 5 canine OS where its expression was suggested to be a potential marker for cisplatin therapy based clinical outcome [36]. This gene may play a role in protecting OS cells, making them less susceptible to the cisplatin where it has been similarly associated with activation of p53 [37,38] and drug resistance in other human tumors [37,39]. Similarly, MGST1 was found to be overexpressed in several human malignant tissues where it was shown to protect cells from several cytotoxic drugs [40,41] as well as by direct detoxification and downstream protection of tumor cells from oxidative stress [40]. Preliminary evidence suggests overexpression of another family member, glutathione S-transferase π, is significantly related to poor histological response to preoperative chemotherapy and poorer prognosis in human OS [41]. Similarly, an *in vitro *model of canine OS (COS31) with high glutathione-S-transferase activity was found to be resistant to cytotoxic effects of cisplatin [42]. Drug resistance issue is of interest in the present study population because despite given postoperative chemotherapy there were dogs unable to extend survival beyond 6 months while others received no chemotherapy and yet lived much longer. This suggests that screening through gene profiles can help with therapeutic decisions by segregating dogs based on key targets for personalized chemotherapy, and hence ensure treatment success.

Genes associated with proliferation, cell cycle and differentiation revealed at present have not been described for OS pathogenesis except for Mortalin (MOT/HSP70), HSP90 and HSP60. MOT/HSP70 has been previously shown to form complexes with p53 tumor suppressor, causing inactivation of the wild type p53 leading to excessive proliferation [43]. Knockdown of MOT/HSP70 by shRNA expression plasmids have proven to cause growth arrest in human OS [44]. Since MOT has been found upregulated in short survivors in this study and since it is known that p53 mutations are common feature of poor prognosis in canine and human OS, extensive in depth investigation should be done to elucidate both the role of MOT/HSP70 as a prognostic marker and its association with p53 in OS. The other two heat shock protein family members revealed by the present study include HSP90 and HSP60, both having been strongly implicated in multiple stages of tumorigenesis from proliferation to impaired apoptosis and angiogenesis, invasion and metastasis. High expressions of HSP90-beta have been reported in 3 human OS cell lines as well as in 3 primary tumors from microarray analysis in previous research [20]. In primary human OS removed at surgery, HSP90α was found to be 40% overexpressed and HSP60 60% [45]. In another, expression of HSP60 was detected in 83% of human OS biopsy specimens, and 43% of these OS patients had increased levels of anti-hsp60 antibodies in their serum [46]. Recently, *in vitro *studies have demonstrated that inhibition of HSP90 exhibits selective cytotoxicity in canine OS cells by downregulation of Met, Akt and p-STAT3, key players for multiple oncogenic signaling pathways [47]. A growing body of evidence suggests that heat shock proteins provide protection to a large group of client proteins: they help protect highly aggressive and metastatic cells from cellular stressors in the tumor microenvironment. The overexpression of all these HSP family members in poor survivors of canine OS makes them interesting in the context of drug development for the next generation of HSP inhibitors.

Another candidate gene of interest to both human and canine OS is NCOR1. We found that NCOR1 was overexpressed in 8 out of 32 (25%) of tumors, all from subgroup B (among the candidates from gene Cluster 1) and there was a clear downregulation among all long survivors. In human OS, NCOR1 was amplified in 22.6% of tumors where it did not correlate with disease free interval, and amplification status correlated significantly with tumor size [48]. The mechanistic role of NCOR1 as an independent negative prognosticator in both human and canine OS is currently unknown, although the overexpression of NCOR1 in 25% of canine tumors could be related to its possible amplification status, similar to what is seen for human OS. Here, we have confirmed the overexpression of MGST1 & NCOR1 in poor survivors as has been previously described for both canine and human OS, and identified two new candidates, ANKRD17 and MRPS31, choosing to validate expression data using quantitative real-time PCR. Those dogs that lived less than 6 months were found to highly express these genes in parallel to the microarray expression data, which suggests these transcripts could be attractive therapeutic targets and markers for future prognosis and disease progression for canine OS.

### Pathway Analyses

Pathway analysis in dogs has its limitations because pathway identification relies heavily on existing functional annotation, which is still limited for the canine species. Our preliminary work networking a limited number of candidate genes identifies c-myc, SP1, p53 and NF-kB as the 'central hubs' connecting the candidate genes, where these transcription factors have been extensively described and associated with poor prognosis in various tumors, including OS [49-51]. Transcripts such as MOT/GRP75, HSP60, p53, HMGB1; VEGFR and NCOR1 from this network have been previously described for cancer pathogenesis. It has been proposed that one or a few key genes can trigger a succession of events leading to abnormal expression of many genes in cancer. Pathway analysis provides a much more practical way to analyze expression data across species which may shed light into common pathways important for targeted therapies. A few pathways have been previously proposed to be of importance in either human or canine OS pathogenesis. Gene enrichment for two major pathways, namely Wnt signaling and chemokine/cytokine signaling, was found to be common among the studies conducted in human OS, and in the present study in dogs. Extensive investigations have been initiated to understand how Wnt signaling is involved in human OS pathogenesis and disease progression. Wnt signaling is not only important for cell proliferation and differentiation, but may also have possible autocrine or paracrine influence on the metastatic potential of OS [52-54]. Preliminary investigations on the roles of Wnt signaling in canine OS pathogenesis and disease progression are underway. Nevertheless, the importance of chemokine signaling has been described in canine OS where its receptor (CXCR4) was found to participate in directional migration *in vitro *[55]. Further research has shown that canine and human primary OS do express CXCR4, which may be involved in metastatic progression of this disease [56]. Molecular strategies targeting these 2 pathways may be beneficial for both human and dogs.

### Use of microarrays for OS prognosis prediction

Clinical, histological and gene expression analysis in combination have the ability to improve the accuracy of clinical diagnostics and prognostics by precisely segregating individuals into meaningful groups, allowing for better decision making for therapeutics. However, in the past, these advanced approaches have, for most studies in human malignancies, failed to be reproduced; instead, parallel studies using distinct data sets frequently identify different gene sets as a result of variations in computational analysis methods to sample processing, array platforms and research questions [57,58]. Other common barriers to reproducibility include differences in experimental design, small sample size and lack of assessment in secondary independent populations. A general problem of prognostic studies is that analyses are carried out on thousands of genes generated from small number of samples, which are often difficult to acquire from dogs where decisions to treat and follow up rely heavily on the owners. Although different human OS gene sets from different platforms and analysis techniques were identified from independent studies, they all seem to achieve agreement when it comes to pathway analysis. This insight is first described in the present study, where we performed gene-pathway enrichment analysis on distinct gene sets from several human OS studies and compared the result with our prognostic genes from the dog to reveal several common pathways conferring pathogenesis and the aggressive phenotype of OS. The conventional single biomarker discovery approach is being slowly overtaken by the idea that complex interactions among multiple genes are required to produce a disease phenotype which can be best described in terms of its deregulation of cellular signaling pathways. Preliminary studies have been initiated in an independent and larger set of canine and human OS samples to further investigate and validate the roles of the main pathways for the aggressive phenotype revealed by the present analysis.

## Conclusion

The premise that gene expression profiling can be used to segregate and then identify negative and favorable prognosis cancer patients is not novel; however this is the first report of its kind in canine OS and probably in any naturally occurring cancer in the dog. The present study has revealed candidate genes that can be followed up in prospective studies as negative prognostic markers and therapeutic targets for canine OS. A molecular-based method to discriminate between short and long survivors of canine OS may be useful for future prognostic stratification of dogs at initial diagnosis, since genes associated with cell cycle/proliferation, drug resistance and metastasis are commonly overexpressed in short survivors of canine OS. In addition, we found that these survival-associated genes enrich prominent pathways such as Wnt and chemokine/cytokine signaling, which were also among the top pathways revealed by comparative pathway analysis with those of human OS gene profiling studies. These findings emphasize the excellent translational opportunity offered by canine expression studies of OS pathogenesis and disease progression that can be used for future therapeutic and prognostic strategies. Other approaches, such as protein profiling as well as *in vitro *studies, will be necessary to elucidate further the biological significance of these findings.

## Methods

### Patient and tumor data

Thirty-two histological confirmed canine OS, with available survival data, that were presented at the University Clinic for Companion Animals in Utrecht The Netherlands from 1996 - 2003 were selected in this study. These dogs were not subjected to any sort of therapy prior to harvesting of the tumor tissue. Tumor samples were harvested under sterile conditions during surgery (amputation/marginal resection/total resection). Samples were snap-frozen in liquid nitrogen and stored in sterile tubes at -70°C. An adjacent tumor specimen was fixed in 4% neutral buffered formalin, decalcified in 10% EDTA and embedded in paraffin. Four μm tissue sections were stained with hematoxylin and eosin where the diagnosis and histological grading was carried out by a board certified veterinary pathologist [59]. Various clinical and pathological parameters were evaluated retrospectively for the dogs included in this study.

### Statistical and survival analysis

All 32 dogs in this study were followed up from the time of diagnosis until death, for up to a period of 5 years. Survival time (ST) was recorded for all dogs. ST was defined as the time period from initial diagnosis until death. Patients were censored if they died due to causes other than metastatic disease. The Kaplan-Meier method was used to draw disease free interval survival curves. Tests for comparison of groups of survival data were made using the Mantel-Cox log rank test (SPSS version 15.0). Univariate Cox proportional hazard analysis was conducted on the overall population of 32 dogs with and without stratification for chemotherapy. Variables found by univariate analysis to influence survival at a cut off of P < 0.15 were further subjected to multivariate analysis using the Cox proportional hazard regression model (backward elimination using Newton Raphson algorithm) to determine whether the variables could independently influence survival in the whole and/or subpopulation of dogs in the present study. Hazard ratios (HR) and confidence intervals (CI) were calculated and reported using EGRET for Windows Version 2.013 (Cytel Software Corporation, Cambridge, MA, USA). Further analyses on discrete variables comparing the subpopulation of dogs were performed using the Fisher's exact test on SPSS version 15.0. Statistical significance was defined as P < 0.05.

### RNA isolation and amplification

Bone tumor samples were pulverized for 45 seconds at a speed of 2000 rounds per minute in the Mikro Dismembrator^® ^U (B. Braun Biotech International, Melsungen, Germany) in ribonuclease free plastic containers. The procedure was repeated if samples were too big. Cooling in liquid nitrogen between cycles was performed. Bone tumor powder was stored in sterile tubes at -70°C. Total RNA isolation and purification was carried out with 600 mU/ml Proteinase K^® ^as an additional pretreatment step using the RNeasy mini kit (Qiagen, The Netherlands) following the manufacturer's protocol. The RNA samples were treated with DNase-I (Qiagen RNase-free DNase kit). Total RNA was quantified using the NanoDrop^® ^spectrophotometer (NanoDrop Technologies). cRNA synthesis was performed using the protocol described. Quality of total and amplified cRNA was analyzed using a bioanalyzer (Agilent Technologies, The Netherlands).

### Microarray hybridization and data normalization

Thirty-two tumors were sorted into two prognosis groups based on survival time (ST). They were defined as short-term survivors (SS; 16 dogs with a negative prognosis that survived less than 6 months) and long-term survivors (LS; 16 dogs with better prognosis that survived 6 months or longer). Microarray hybridization, scanning and image analysis were conducted according to the protocol described [60]. A common reference cRNA pool consisting of all 32 tumors was used to hybridize against each tumor on the array. Dyes were swapped as described in previous literatures [61], where equal numbers of samples within each tumor group were subjected to dye swap to avoid dye bias effect. Defective spots were flagged and normalization was carried out using the Lowess print-tip normalization technique. Log transformation was applied to the microarray raw data after normalization and data from dye swap arrays were switched before they were subjected for further statistical analysis. The microarray data files were deposited in the public database (GEO: GSE14033).

### Microarray data analysis

Microarray data analysis and calculations were performed using a two class unpaired approach to compare the normalized and log-transformed expression data between the two groups. Calculations were done using *Significance Analysis of Microarray *(SAM) (Stanford University, CA, USA) [62], using 100 permutations to the k-nearest neighbor with 10 neighbors and newly initialized random seeds for each analysis. The gene list for this analysis was generated at a false discovery rate (FDR) of 10%. Genes with fold changes of 1.4 and above were selected for further analyses [63]. Hierarchical clustering of genes that were differentially regulated between the two groups of tumors was performed. A two-dimensional dendrogram of both gene tree and condition tree based on standard correlation was generated using the GeneSpring^® ^(Agilent Technologies, The Netherlands) software [64]. The Gene Ontology (GO) database was used to cross check the gene molecular and biological functions .

### Quantitative real-time PCR

Total tumor RNA was isolated using the protocol mentioned above. Synthesis of cDNA was carried out from 1.0 μg total RNA in 40 μl reaction volumes using the iScript™ cDNA synthesis kit as described by the manufacturer's protocol (Bio-Rad, The Netherlands). Quantitative real-time PCR (Q-PCR) was performed on four candidate genes from the list of differential expressed genes, as well as the ribosomal protein S19 (RPS19) and hypoxanthine phosphoribosyl transferase (HPRT) as endogenous reference gene for normalizations. Primer sets (Table 6) were designed using the software Primer 3 [65]. Q-PCR was carried out using the SYBR^® ^green fluorescent dye method, which was further analyzed by the Bio-Rad MyIQ software (BioRad, The Netherlands). The Q-PCR products were sequenced to verify the specificity of the primer sets. Data was analyzed on REST-XL (pair wise fixed reallocation and randomization test) software [66] that estimates a relative normalized fold change of gene expression between the two groups which is then compared to the microarray fold change data.

**Table 6:** Primers used for quantitative real-time PCR

**Gene symbol**	**Forward and Reverse Primers**	**Temp (°C)**
ANKRD17	FW: 5'-AAGTAGCGCACCACCTTCAC-3'	60.0
	RW: 5'-CTAGCAGCAAATGGTGGACA-3'	
		
MRPS31	FW: 5'-GAATTGGTCCTTGCTTTGGA-3'	60.0
	RW: 5'-ATCCAGTGGACGAAAGATGG-3'	
		
NCOR1	FW: 5'-TCTTCCTCTGCGTTTTCCAT-3'	59.6
	RW: 5'-GCATCCCAAAAACTTTGGAC-3'	
		
MGST1	FW: 5'-CGGACAGATGATAGGGTGG-3'	62.0
	RW: 5'-GATTTGGCTGGGGAAGG-3'	
		
RPS19	FW: 5'-CCTTCCTCAAAAAGTCTGGG-3'	61.0
	RW: 5'-GTTCTCATCGTAGGGAGCAAG-3'	
		
HPRT	FW: 5'-AGCTTGCTGGTGAAAAGGAC-3'	56.0
	RW: 5'-TTATAGTCAAGGGCATATCC-3'	

### Pathway analyses

Analysis of gene regulatory networks present among the differentially expressed genes identified by SAM was performed using the 'Shortest Path Analysis' algorithm of MetaCore™ Analytical Suite (GeneGo Inc., St. Joseph, MI, USA) [67]. In addition, for further insight into overall pathway discovery, we applied a less stringent rule where correction for multiple testing method was not applied in obtaining differential expressed genes [68]. EDGE (Extraction of Differential Gene Expression) software  was used to generate the genes differentially expressed between the short and long survivors of canine OS at a cutoff threshold of P < 0.05 [69]. The differential expressed genes identified by EDGE from the present study in canine were compared with the human homologues to obtain a list of gene symbols or human RefSeq ID that was later subjected for pathway analysis using the program PANTHER^® ^(**P**rotein **AN**alysis **TH**rough **E**volutionary **R**elationships) [60,68]. Pathways revealed by this analysis were sorted according to number of gene hits and compared with the top 20 pathways from various human OS microarray studies.

## Abbreviations

SS: short survivors; LS: long survivors; ST: overall survival time; DFI: disease free interval; FDR: false discovery rate; SAM: Significance Analysis of Microarray; GEO: Gene Expression Omnibus; QPCR: Quantitative Real Time PCR; CI: Confidence Interval

## Competing interests

The authors declare that they have no competing interests.

## Authors' contributions

GTS analyzed the clinicopathological and microarray data, performed statistical analyses, designed primers, carried out QPCR, pathway analyses, drafted the manuscript and revised the manuscript according to the reviewers' recommendations. MEW helped with RNA amplification, microarray hybridizations and contributed in the research discussions. JAM helped with assisted in coordination of work, data interpretation, pathway analyses and final manuscript revision. JK participated in the experimental design, set up of the initial study, carried out the overall responsibility of the research performed and revised the manuscript critically. NASR assisted with the bioinformatics aspect of microarray analysis, contributed in research discussions and reviewed the manuscript critically. HF assisted in sample collection, retrieval of survival data and performed RNA isolation. All authors have reviewed and approved the final and revised manuscript.

## Supplementary Material

Additional file 1 **Univariate analysis**. a) Univariate analysis of specific variable influences on survival time (ST) among dogs from the entire population of study. b) Variables with P < 0.15 from univariate analysis that were subsequently forced into multivariate model identifies elevation of serum alkaline phosphatase with significantly increased HR for a shorter ST. **§ **Missing data, * Category which was used as baseline reference, ‡ Continuous variables Click here for file

Additional file 2 **Cox proportional hazard analysis upon stratification for postoperative chemotherapy**. Cox proportional hazard analysis (univariate) upon stratification for postoperative chemotherapy revealed no significant influence of the variables assessed on survival time of the dogs in the total population of study (n = 32). Click here for file

Additional file 3 **Fisher's exact test and univariate Cox proportional hazard analysis carried out on the new subgroups**. Both subgroups did not differ in frequency distribution of the variables assessed and none of the variables assessed were found to significantly influence survival.
NA, non applicable; HR, hazard ratio Click here for file

Additional file 4 **Differential expressed genes categorized based on their molecular functions**. Click here for file
