# Supplementary material for: Gene expression profiling of canine osteosarcoma reveals genes associated with short and long survival times
Source: Mol Cancer. 2009 Sep 7;8:72. doi: 10.1186/1476-4598-8-72 (PMC2746177; doi:10.1186/1476-4598-8-72)
Supplement: Additional file 1 — Univariate analysis. a) Univariate analysis of specific variable influences on survival time (ST) among dogs from the entire population of study. b) Variables with P < 0.15 from univariate analysis that were subsequently forced into multivariate model identifies elevation of serum alkaline phosphatase with significantly increased HR for a shorter ST. § Missing data, * Category which was used as baseline reference, ‡ Continuous variables [file 1476-4598-8-72-S1.doc]

| **Parameter** | **No of dogs (n)** | **Hazard Ratio** | **Lower CI** | **Upper CI** | **P value** |
| --- | --- | --- | --- | --- | --- |
| **Age** | 32 | 1.137 | 0.956 | 1.353 | 0.147 |
| **Gender** |  | 1.438 | 0.631 | 3.273 | 0.387 |
| Male* | 18 |  |  |  |  |
| Female | 14 |  |  |  |  |
| **Neuter status** |  | 1.325 | 0.551 | 3.191 | 0.53 |
| Neutered* | 11 |  |  |  |  |
| Non neutered | 21 |  |  |  |  |
| **AP §** | 23 | 1.003 | 1 | 1.007 | 0.078 |
| **Histo grade** |  | 1.185 | 0.466 | 3.012 | 0.722 |
| Low and medium* | 7 |  |  |  |  |
| High | 25 |  |  |  |  |
| **Postoperative Chemotherapy** |  | 1.590 | 0.720 | 3.511 | 0.251 |
| Non treated* | 11 |  |  |  |  |
| Treated | 21 |  |  |  |  |

**Additional file 1**

**a)**

**b)**

| **Variable** | **Hazard ratio** | **95% CI** | **P value** |
| --- | --- | --- | --- |
| **AP ^§^** ‡ | 1.005 | 1.000-1.009 | **0.035** |
| **Age** ‡ | 1.18 | 0.915-1.522 | 0.202 |
