# Supplementary material for: Gene expression profiling of canine osteosarcoma reveals genes associated with short and long survival times
Source: Mol Cancer. 2009 Sep 7;8:72. doi: 10.1186/1476-4598-8-72 (PMC2746177; doi:10.1186/1476-4598-8-72)
Supplement: Additional file 2 — Cox proportional hazard analysis upon stratification for postoperative chemotherapy. Cox proportional hazard analysis (univariate) upon stratification for postoperative chemotherapy revealed no significant influence of the variables assessed on survival time of the dogs in the total population of study (n = 32). [file 1476-4598-8-72-S2.doc]

**Additional file 2**

|  |  | **with chemotherapy stratification** | | | |  |
| --- | --- | --- | --- | --- | --- | --- |
| **Parameter** | **No of dogs (n)** | **Hazard Ratio** | **Lower CI** | **Upper CI** | **P value** |  |
|  |  |  |  |  |  |  |
| **Age** | 32 | 1.118 | 0.9393 | 1.3320 | 0.200 |  |
| **Gender** |  | 0.623 | 0.2554 | 1.5200 | 0.298 |  |
| Male | 18 |  |  |  |  |  |
| Female | 14 |  |  |  |  |  |
| **Neuter status** |  | 0.724 | 0.2747 | 1.9090 | 0.514 |  |
| Neutered | 11 |  |  |  |  |  |
| Non neutered | 21 |  |  |  |  |  |
| **AP ^§^** | 23 | 1.003 | 0.9994 | 1.0070 | 0.153 |  |
| **Histo grade** |  | 0.928 | 0.3638 | 2.3680 | 0.877 |  |
| Low and medium | 7 |  |  |  |  |  |
| High | 25 |  |  |  |  |  |
|  |  |  |  |  |  |  |
| **§** missing data | | | | | | |
