# Supplementary material for: Gene expression profiling of canine osteosarcoma reveals genes associated with short and long survival times
Source: Mol Cancer. 2009 Sep 7;8:72. doi: 10.1186/1476-4598-8-72 (PMC2746177; doi:10.1186/1476-4598-8-72)
Supplement: Additional file 3 — Fisher's exact test and univariate Cox proportional hazard analysis carried out on the new subgroups. Both subgroups did not differ in frequency distribution of the variables assessed and none of the variables assessed were found to significantly influence survival. NA, non applicable; HR, hazard ratio [file 1476-4598-8-72-S3.doc]

**Additional file 3**

| **Variable** | **Univariate Cox regression analysis** | | | | **(Fisher's exact test)** |
| --- | --- | --- | --- | --- | --- |
|  | **8A subgroup** | **HR (CI) for subgroup 8A** | **8B subgroup** | **HR (CI) for subgroup 8B** |  |
|  | **(n)** | **P value** | **(n)** | **P value** | **P value** |
|  |  |  |  |  |  |
| **Gender** |  | 0.003 (0.000-646.96) |  | 0.006 (0.00- 39.93) | > 0.999 |
| Male | 5 | 0.351 | 4 | 0.256 |  |
| Female | 3 |  | 4 |  |  |
| **Neuter status** |  | 0.355 (0.070-1.810) |  | 3.499 (0.485-25.272) | > 0.999 |
| Neutered | 3 | 0.213 | 2 | 0.214 |  |
| Intact | 5 |  | 6 |  |  |
| **Postoperative Chemotherapy** |  | 3.465 (0.620-19.354) |  | 0.963 (0.206-4.503) | 0.614 |
| No | 4 | 0.157 | 3 | 0.962 |  |
| Yes | 4 |  | 5 |  |  |
| **Histological grade** |  | 0.780 (0.088 - 6.875) |  | 2.248 (0.371-13.622) | > 0.999 |
| Low and medium | 1 | 0.823 | 2 | 0.378 |  |
| High | 7 |  | 6 |  |  |
| **AP** | 5 | 0.995 (0.984 - 1.007) | 5 | 0.999 (0.994-1.004) | - |
|  |  | 0.441 |  | 0.749 |  |
