# Supplementary material for: Gene expression profiling of canine osteosarcoma reveals genes associated with short and long survival times
Source: Mol Cancer. 2009 Sep 7;8:72. doi: 10.1186/1476-4598-8-72 (PMC2746177; doi:10.1186/1476-4598-8-72)
Supplement: Additional file 4 — Differential expressed genes categorized based on their molecular functions. [file 1476-4598-8-72-S4.doc]

**Additional file 4**

| **Gene ID** | **Fold change** | **Gene description** | **Gene symbol** |
| --- | --- | --- | --- |
|  |  |  |  |
| **DNA repair and integrity** | | | |
| DG2-18m22 | 4.6 | plasma glutamate carboxypeptidase | PGCP |
| DG2-72g4 | 2.7 | ankyrin repeat domain protein 17 isoform a | ANKRD17 |
| DG2-112n11 | 2.1 | Kinesin heavy chain (Ubiquitous kinesin heavy chain) | UKHC |
| DG2-123a3 | 2.0 | Microsomal glutathione S-transferase 1 | MGST1 |
| DG32-161c11 | 2.0 | WNK lysine deficient protein kinase 1 | WNK1 |
| DG14-71c7 | 1.8 | serine/arginine repetitive matrix 1 | SRRM1 |
| DG14-14i19 | 1.7 | Stress-70 protein, mitochondrial precursor (HSAP9) (GRP 75) (Mortalin) | MOT |
| DG2-63l7 | 1.7 | nuclear receptor co-repressor 1 | NCOR1 |
| DG32-237k11 | 1.6 | Ribosomal L1 domain containing protein 1(PBK1 protein) | RSL1D1 |
| DG2-72p3 | 1.6 | Vacuolar ATP synthase subunit C | V-ATPase C |
| DG11-239n21 | 1.6 | cell-cycle and apoptosis regulatory protein 1 | CCAR1 |
| DG2-94j4 | 1.5 | high-mobility group box 1 | HMGB1 |
| DG2-106f2 | 1.4 | Translocation protein SEC63 homolog | SEC63 |
| DG2-25k5 | 1.4 | SMC6 protein | SMC6 |
| DG11-243e16 | 1.4 | splicing factor, arginine/serine-rich 2, interacting protein | SFRS2IP |
|  |  |  |  |
| **Cell cycle/ proliferation** | | | |
| DG2-23c15 | 3.3 | cofilin 2 | CFL2 |
| DG32-161c11 | 2.0 | WNK lysine deficient protein kinase 1 | WNK1 |
| DG14-71c7 | 1.8 | serine/arginine repetitive matrix 1 | SRRM1 |
| DG14-14i19 | 1.7 | Stress-70 protein, mitochondrial precursor (HSAP9) (GRP 75) (Mortalin) | MOT |
| DG32-237k11 | 1.6 | Ribosomal L1 domain containing protein 1(PBK1 protein) | RSL1D1 |
| DG11-239n21 | 1.6 | cell-cycle and apoptosis regulatory protein 1 | CCAR1 |
| DG2-106f2 | 1.4 | Translocation protein SEC63 homolog | SEC63 |
| DG2-25k5 | 1.4 | SMC6 protein | SMC6 |
|  |  |  |  |
| **Stress response** | | | |
| DG2-90b10 | 2.2 | Heat shock protein HSP 90-alpha | HSP86 |
| DG42-128j23 | 2.1 | paraoxonase 1 | PON1 |
| DG2-123a3 | 2.0 | Microsomal glutathione S-transferase 1 | MGST1 |
| DG32-161c11 | 2.0 | WNK lysine deficient protein kinase 1 | WNK1 |
| DG14-14i19 | 1.7 | Stress-70 protein, mitochondrial precursor (HSAP9) (GRP 75) (Mortalin) | MOT |
| DG32-216j13 | 1.6 | Flavin reductase (FR) (NADPH-dependent diaphorase) (FLR) | BVRB |
| DG2-24i24 | 1.4 | 60 kDa heat shock protein, mitochondrial precursor | HSP60 |
|  |  |  |  |
| **Apoptosis regulation** | | | |
| DG2-90b10 | 2.2 | Heat shock protein HSP 90-alpha | HSP86 |
| DG14-14i19 | 1.7 | Stress-70 protein, mitochondrial precursor (HSAP9) (GRP 75) (Mortalin) | MOT |
| DG2-72p3 | 1.6 | Vacuolar ATP synthase subunit C | V-ATPase C |
| DG11-239n21 | 1.6 | cell-cycle and apoptosis regulatory protein 1 | CCAR1 |
| DG2-94j4 | 1.5 | high-mobility group box 1 | HMGB1 |
| DG2-24i24 | 1.4 | 60 kDa heat shock protein, mitochondrial precursor | HSP60 |
| **continued** |  |  |  |
| **Gene ID** | **Fold change** | **Gene description** | **Gene symbol** |
|  |  |  |  |
| **Protein modification and metabolism** | | |  |
| DG2-18m22 | 4.6 | plasma glutamate carboxypeptidase | PGCP |
| DG2-21g13 | 4.1 | WD repeat and SOCS box containing protein 2 | WSB2 |
| DG2-72g4 | 2.7 | ankyrin repeat domain protein 17 isoform a | ANKRD17 |
| DG2-90b10 | 2.2 | Heat shock protein HSP 90-alpha | HSP86 |
| DG2-123a3 | 2.0 | Microsomal glutathione S-transferase 1 | MGST1 |
| DG14-71c7 | 1.8 | serine/arginine repetitive matrix 1 | SRRM1 |
| DG14-14i19 | 1.7 | Stress-70 protein, mitochondrial precursor (HSAP9) (GRP 75) (Mortalin) | MOT |
| DG2-63l7 | 1.7 | nuclear receptor co-repressor 1 | NCOR1 |
| DG32-237k11 | 1.6 | Ribosomal L1 domain containing protein 1(PBK1 protein) | RSL1D1 |
| DG2-72p3 | 1.6 | Vacuolar ATP synthase subunit C | V-ATPase C |
| DG32-216j13 | 1.6 | Flavin reductase (FR) (NADPH-dependent diaphorase) (FLR) | BVRB |
| DG2-94j4 | 1.5 | high-mobility group box 1 | HMGB1 |
| DG2-106f2 | 1.4 | Translocation protein SEC63 homolog | SEC63 |
| DG2-25k5 | 1.4 | SMC6 protein | SMC6 |
| DG2-24i24 | 1.4 | 60 kDa heat shock protein, mitochondrial precursor | HSP60 |
| DG11-243e16 | 1.4 | splicing factor, arginine/serine-rich 2, interacting protein | SFRS2IP |
|  |  |  |  |
| **mRNA transcription regulation** | | |  |
| DG2-18m22 | 4.6 | plasma glutamate carboxypeptidase | PGCP |
| DG2-21g13 | 4.1 | WD repeat and SOCS box containing protein 2 | WSB2 |
| DG2-72g4 | 2.7 | ankyrin repeat domain protein 17 isoform a | ANKRD17 |
| DG2-90b10 | 2.2 | Heat shock protein HSP 90-alpha | HSP86 |
| DG32-161c11 | 2.0 | WNK lysine deficient protein kinase 1 | WNK1 |
| DG14-71c7 | 1.8 | serine/arginine repetitive matrix 1 | SRRM1 |
| DG2-63l7 | 1.7 | nuclear receptor co-repressor 1 | NCOR1 |
| DG32-237k11 | 1.6 | Ribosomal L1 domain containing protein 1(PBK1 protein) | RSL1D1 |
| DG2-94j4 | 1.5 | high-mobility group box 1 | HMGB1 |
| DG2-106f2 | 1.4 | Translocation protein SEC63 homolog | SEC63 |
| DG2-24i24 | 1.4 | 60 kDa heat shock protein, mitochondrial precursor | HSP60 |
|  |  |  |  |
